# Perceptions, Knowledge, and Behaviors Related to COVID-19 Among Social Media Users: Cross-Sectional Study

**DOI:** 10.2196/19913

**Published:** 2020-09-08

**Authors:** Khawla F Ali, Simon Whitebridge, Mohammad H Jamal, Mohammad Alsafy, Stephen L Atkin

**Affiliations:** 1 Royal College of Surgeons in Ireland-Medical University of Bahrain Adliya Bahrain; 2 Department of Surgery, Kuwait University Kuwait; 3 Clinica Dental Center Kuwait

**Keywords:** COVID-19, social media, public health, perception, knowledge, health information, health education, virus

## Abstract

**Background:**

Social media is one of the most rapid and impactful ways of obtaining and delivering information in the modern era.

**Objective:**

The aim of this study was to rapidly obtain information on public perceptions, knowledge, and behaviors related to COVID-19 in order to identify deficiencies in key areas of public education.

**Methods:**

Using a cross-sectional study design, a survey web link was posted on the social media and messaging platforms Instagram, Twitter, and WhatsApp by the study investigators. Participants, aged ≥18 years, filled out the survey on a voluntary basis. The main outcomes measured were knowledge of COVID-19 symptoms, protective measures against COVID-19, and source(s) of information about COVID-19. Subgroup analyses were conducted to determine the effects of age, gender, underlying illness, and working or studying in the health care industry on the perceived likelihood of acquiring COVID-19 and getting vaccinated.

**Results:**

A total of 5677 subjects completed the survey over the course of 1 week. “Fever or chills” (n=4973, 87.6%) and “shortness of breath” (n=4695, 82.7%) were identified as the main symptoms of COVID-19. Washing and sanitizing hands (n=4990, 87.9%) and avoiding public places and crowds (n=4865, 85.7%) were identified as the protective measures most frequently used against COVID-19. Social media was the most utilized source for information on the disease (n=4740, 83.5%), followed by the World Health Organization (n=2844, 50.1%). Subgroup analysis revealed that younger subjects (<35 years), males, and those working or studying in health care reported a higher perceived likelihood of acquiring COVID-19, whereas older subjects, females, and those working or studying in non–health care areas reported a lower perceived likelihood of acquiring COVID-19. Similar trends were observed for vaccination against COVID-19, with older subjects, females, and those working or studying in non–health care sectors reporting a lower likelihood of vaccinating against COVID-19.

**Conclusions:**

Our results are indicative of a relatively well-informed cohort implementing appropriate protective measures. However, key knowledge deficiencies exist with regards to vaccination against COVID-19, which future efforts should aim at correcting.

## Introduction

The novel coronavirus SARS-CoV-2 has been at the core of the devastating COVID-19 pandemic. The pandemic, which originated from Hubei Province, China, has spread around the globe, having claimed 512,842 lives as of July 2, 2020 [1,2]. 

COVID-19 is predominately characterized by systemic symptoms, such as fever and fatigue, and respiratory symptoms, such as cough, expectoration, and a runny nose [3,4]. In a recent analysis examining the relationship between symptoms of COVID-19 and disease severity, fatigue and expectoration were found to be the most critical, positive prognostic symptoms of COVID-19 severity, whereas a runny nose and nausea were favorable prognostic factors [4]. Transmission of SARS-CoV-2 occurs primarily via direct contact with the respiratory droplets of infected individuals, or via indirect contact with virally contaminated objects [5]. Increasing evidence suggests asymptomatic carriers have the ability to transmit the virus, making it ever so critical to identify and isolate these individuals effectively and rapidly [5-7]. Mortality in patients with COVID-19 differs across countries and has been significantly associated with age and comorbidities in subjects, ranging from 1.9% among ambulatory, low-risk patients to 21.7% among hospitalized, higher-risk individuals [8].

To date, despite intensive medical research on the RNA (ribonucleic acid) of the virus, no treatment has been found for COVID-19; only supportive measures are being used for those who need critical care. Public preventative measures remain key for slowing down its spread. One of the most effective methods for slowing or halting the spread of COVID-19 has been social distancing, and in some instances, social isolation [9,10]. Moreover, hygienic practices such as frequent hand washing, hand sanitizing, and wearing face masks, if implemented widely and correctly, can aid in reducing the spread of the virus [10]. Such simple yet effective measures mandate powerful messages, displayed on widely viewed communication platforms, for impactful dissemination. Additionally, with the rapidly evolving situation surrounding COVID-19, the speed of information dissemination is critical.

One of the fastest and most accessible platforms for broadcasting information is social media. Social media represents a conglomerate of electronic platforms utilized for creating and sharing information, ideas, messages, etc. Such platforms include social networking websites such as Twitter, Instagram, and Facebook, and messaging platforms like WhatsApp. These outlets have had a major global impact, with billions of users worldwide. Their use has extended from personal territories to organizational utilization to spread reliable information. Such organizations include the World Health Organization (WHO) and Centers of Disease Control and Prevention (CDC), both posting daily updates about the current pandemic, and each registering hundreds of thousands of followers worldwide.

The COVID-19 pandemic requires not only rapid information spread but also information identification and collation. This is essential for identifying gaps and misconceptions in the public’s knowledge (“fake news”) and behaviors toward the novel coronavirus. Social media offers an outlet to address both. Although the utilization of such platforms as tools to undertake research is in its infancy, their speed and extensive reach adds a unique digital print to the field of cross-sectional research [11,12]. Therefore, the aim of this cross-sectional study is to examine, through several social media platforms, the public’s perceptions, knowledge, and behaviors related to the current COVID-19 pandemic to identify deficiencies in public education.

## Methods

### Study Population

Users of the social media and messaging platforms Instagram, Twitter, and WhatsApp, who were aged ≥18 years, were recruited to participate in the survey via a web link. The link was posted on the public social media pages of the following authors KFA, MHJ, and MA. Adult users who viewed the authors’ public pages were asked to voluntarily fill out the survey. Resharing of the survey link by users was permitted on all three platforms for snowball sampling. The survey link was active from March 28 to April 4, 2020. Users with the highest visibility of the authors’ accounts resided predominantly in the Arabian Gulf countries: Bahrain, Kuwait, Saudi Arabia, and United Arab Emirates. The survey was administered in both English and Arabic, the two predominantly spoken languages in these countries.

### Survey Administration

Our survey was composed of 13 questions (see Multimedia Appendix 1 for questions). The first 7 questions addressed topics such as source(s) of information on COVID-19, viral preventative behavior(s), and knowledge of symptoms of COVID-19. We also asked whether the participant had acquired the infection; if they answered “no,” they were asked to state their perceived likelihood of acquiring it in the next 3 months. Finally, we asked participants whether they would vaccinate against COVID-19. The other 6 questions pertained to subject demographics: age group, gender, educational level, country of current residence, underlying illness(es), and if the participant works or studies in the health care sector. The survey questions were partially adapted from a questionnaire designed and published by the Understanding America Study, which is maintained by the Center for Economic and Social Research at the University of Southern California [13]. The survey was designed on the online platform Zoho Creator (Zoho Corp). Informed consent in the form of agreement to an information leaflet was obtained from all subjects prior to survey initiation. The survey overall took less than 5 minutes to complete. The study was approved by the Research Ethics Committee at the Royal College of Surgeons in Ireland-Medical University of Bahrain.

### Data Collection and Analysis

All data on personal demographics (age groups, gender, highest educational level, working or studying in the health care sector, presence of medical comorbidities, country of current residence, and status of COVID-19 infection) were categorically expressed as counts and percentages of total respondents. Data on source(s) of information during COVID-19, viral preventative behavior(s), and knowledge of symptoms of COVID-19 were also expressed as counts and percentages of total respondents.

Subgroup categorical analyses were conducted to examine the effects of age (≥35 years versus <35 years), gender (female versus male), underlying illness(es) (yes versus no), and status of working or studying in the health care industry (yes versus no) on the perceived likelihood of being infected with COVID-19 in the next 3 months, as well as the likelihood of vaccinating against COVID-19. Results were categorically expressed as counts and percentages of total respondents in each of the subgroups.

### Patient and Public Involvement

The development of the research questions and outcomes was primarily executed by the study investigators and guided by current literature. Patients were not involved in the design of this study. The involvement of patients and the public, however, was crucial during the recruitment phase of the study (ie, dissemination of the survey link, via snowballing effect, through users’ social media accounts to others in the digital community). After publication, the results will be disseminated to participants on the same platforms that hosted the survey link, through a short, interactive video recorded and posted by the principle investigator of the study.

## Results

A total of 5677 subjects completed the survey. Of the survey respondents, 3945 (69.5%) were female, 3737 (65.9%) were <35 years old, and 4257 participants (75.0%) reported a higher educational level. Amongst respondents, 1250 (22%) reported working or studying in the health care industry and 4003 (70.5%) reported no underlying illness. The majority of respondents resided in Bahrain (3179/5677, 56.0%), Kuwait (784/5677, 13.8%), and Saudi Arabia (693/5677, 12.2%). Only 55 respondents (1%) reported that they had been diagnosed with COVID-19. Other baseline demographics are detailed in Table 1.

**Table 1:** Baseline characteristics of the study population (N=5677).

Characteristic			Participants
Female, n (%)			3945 (69.5)
**Age, n (%)**			
	18-24 years	1712 (30.2)	
	25-34 years	2025 (35.7)	
	35-44 years	1104 (19.4)	
	45-54 years	534 (9.4)	
	55-64 years	256 (4.5)	
	≥65 years	46 (0.8)	
**Educational status, n (%)**			
	Primary school	11 (0.2)	
	Intermediate school	68 (1.2)	
	High school	1341 (23.6)	
	College/higher education	4257 (75.0)	
**Work or study in the health care sector, n (%)**			
	Yes	1250 (22.0)	
	No	4427 (78.0)	
**Medical problems, n (%)**			
	I have no medical problems	4003 (70.5)	
	I have medical problems	1674 (29.5)	
**Type of medical problems^a^, n (%)**			
	High blood pressure	387 (23.1)	
	Diabetes	362 (21.6)	
	Heart disease	74 (4.4)	
	Lung disease	198 (11.8)	
	Cancer	31 (1.9)	
	Other	946 (56.5)	
**Country/region of current residence, n (%)**			
	Bahrain	3179 (56.0)	
	Kuwait	784 (13.8)	
	Saudi Arabia	693 (12.2)	
	United Arab Emirates	324 (5.7)	
	Oman	232 (4.1)	
	Qatar	71 (1.3)	
	Other Arab countries	166 (2.9)	
	Asian countries (excluding Arab countries)	43 (0.8)	
	Europe	118 (2.1)	
	North America	58 (1.0)	
	South America	2 (0)	
	Australia and New Zealand	7 (0.1)	
**COVID-19 diagnosis, n (%)**			
	Yes	55 (1.0)	
	No	5622 (99.0)	

^a^n=1674.

**Figure 1:**
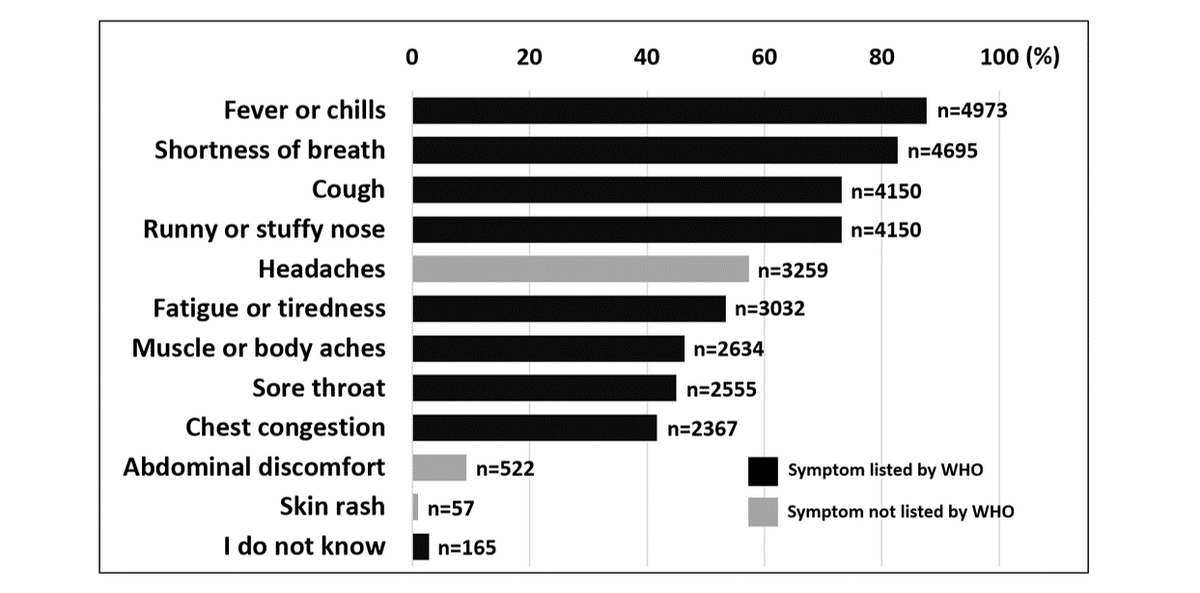
Knowledge about main symptoms of COVID-19 infection. Results are expressed as % of respondents. n=5,677.

**Figure 2:**
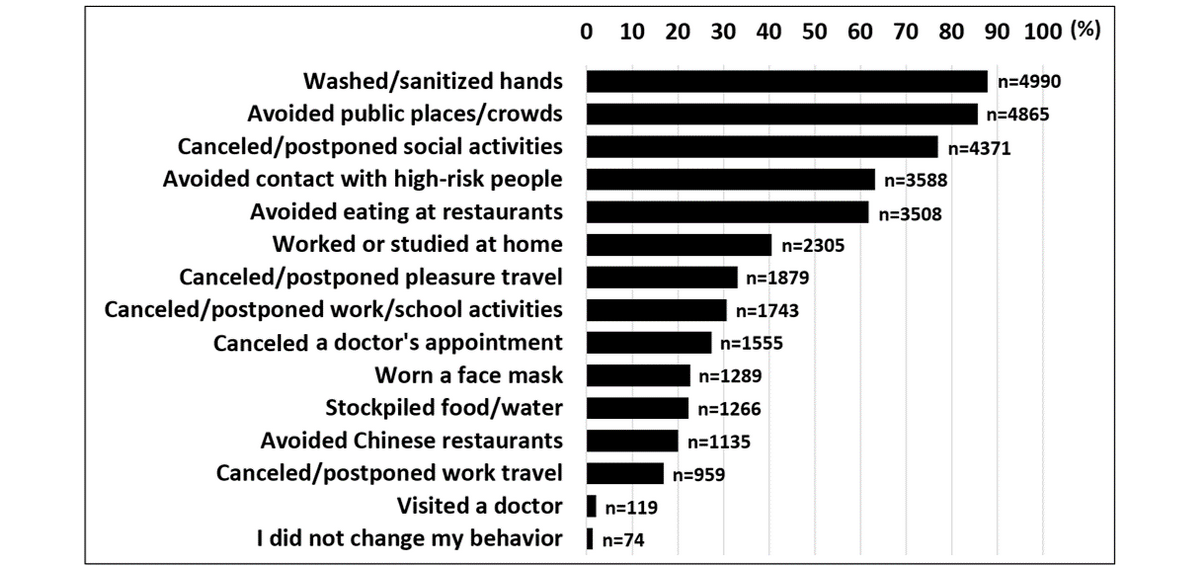
Behaviour(s) done in past week to prevent COVID-19 infection. Results are expressed as % of respondents. n=5,677.

**Figure 3:**
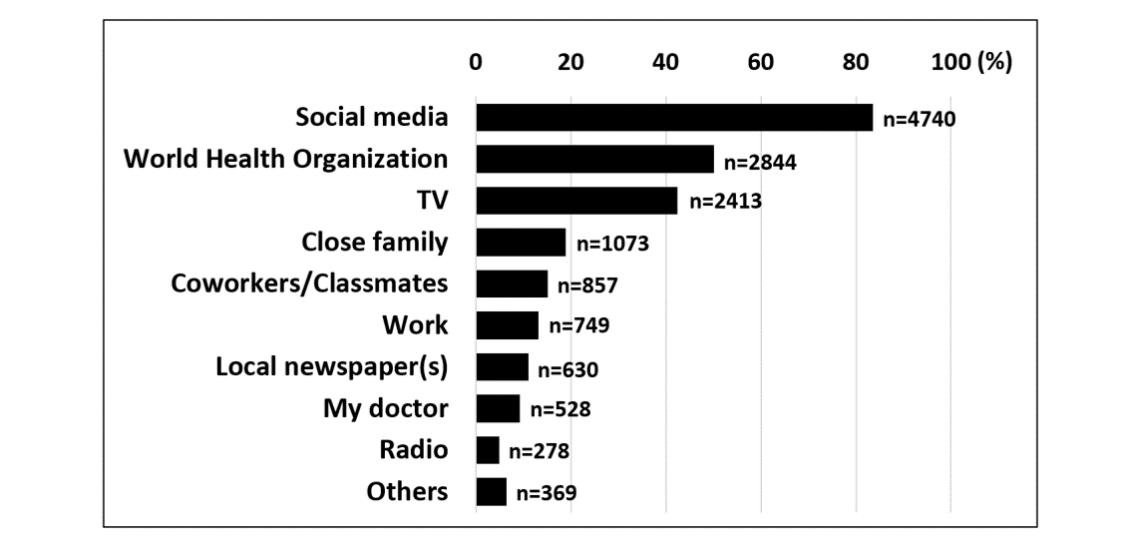
Source(s) of COVID-19 information. Results are expressed as % of respondents (N=5677).

The majority of respondents identified “fever or chills” (4973/5677, 87.6%), “shortness of breath” (4695/5677, 82.7%) and “cough” (4150/5677, 73.1%) as the main symptoms of COVID-19 (Figure 1). Only 165 respondents (2.9%) reported “I do not know” when asked about the main symptoms of COVID-19 (Figure 1). The most reported preventative behaviors were frequent washing and sanitizing hands (4990/5677, 87.9%), avoiding public places and crowds (4865/5677, 85.7%), and canceling or postponing social activities (4371/5677, 77.0%) (Figure 2). Only 74 subjects (1.3%) reported not changing their behavior in response to COVID-19 (Figure 2). The most utilized sources for COVID-19 information were social media platforms (4740/5677, 83.5%), the WHO (2844/5677, 50.1%), and TV (2413/5677, 42.5%) (Figure 3).

**Figure 4:**
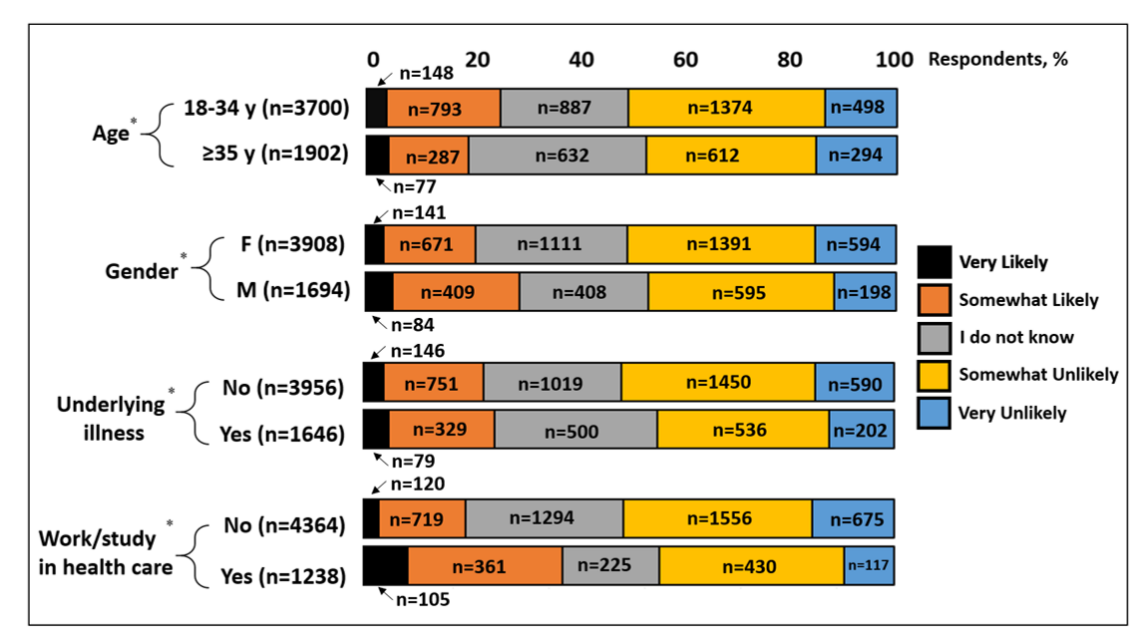
Perceived likelihood of acquiring COVID-19 infection in the next 3 months. Results are categorized by age group, gender, presence of underlying illness(es), and if the subject works or studies in the health care industry. Results are expressed as % of respondents. *n=5602 in all categories (55 subjects reporting COVID-19 infection were eliminated from this analysis; 20 subjects with no response were also eliminated).

**Figure 5:**
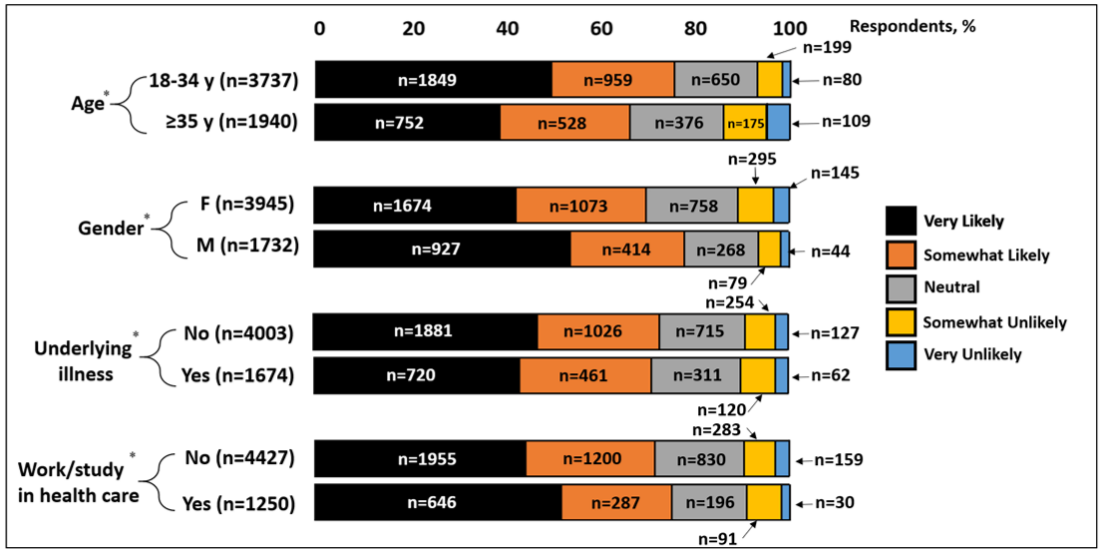
Likelihood of vaccinating against COVID-19. Results are categorized by age group, gender, presence of underlying illness and if subject works/studies in healthcare industry. Results are expressed as % of respondents. *n=5,677 in each category.

In a subgroup analysis examining the effects of age, gender, underlying illnesses, and work or study in the health care sector on the perceived likelihood of acquiring COVID-19 in the next 3 months, younger subjects (<35 years), male subjects, and health care workers and students reported a higher likelihood of acquiring COVID-19, expressed as “very likely” and “somewhat likely” (Figure 4). In a secondary subgroup analysis investigating the effects of the latter on the likelihood of vaccinating against COVID-19, younger subjects (<35 years), male subjects, and those working or studying in health care also reported a higher likelihood of vaccinating, expressed as “very likely” and “somewhat likely” (Figure 5).

## Discussion

### Principal Findings

In the midst of an unprecedented global health crisis, it is critical to know if the educational messages for the prevention of COVID-19 are being delivered, understood, and implemented by the public. Our survey, conducted through three social media outlets, facilitated a large and rapid collection of 5677 responses within 7 days and showed that the majority of respondents were well aware of the symptoms of COVID-19 and the measures necessary to prevent it, as per the WHO guidelines [14]. Additionally, a large portion of subjects (around 50%) utilized reliable sources of information such as the WHO. Such collective responses are indicative of a well-informed cohort, which may be explained by the younger, more educated (75% had higher education) users of social media. It is of note that a large number of these responses (56.0%) were from people in Bahrain. The spread of COVID-19 in Bahrain was well contained at the time of survey distribution (998 cases as of April 11, 2020) [2]. The number of fatalities (7 as of April 11, 2020) [2] has been very low considering it is the third most densely populated country in the world. Such statistics can be due to the early and extensive communication with the public, particularly through social media outlets [15,16]; this highlights the importance of conveying general knowledge about the virus to residents to control spread.

Our subgroup analysis examining the effects of age (<35 versus ≥35 years old), gender, presence of underlying illnesses, and work or study in health care revealed some intriguing findings. Younger subjects (<35 years old) reported a higher likelihood of vaccinating against COVID-19 compared to older participants. This may be a reflection of their higher perceived likelihood of acquiring COVID-19, as demonstrated in our subgroup analysis. A higher perceived likelihood of infection may be explained by higher chances of coming into contact with perceived high-risk groups as mandated by work, social, or study environments. Similarly, male subjects reported a higher likelihood of acquiring the infection and vaccination than their female counterparts. This may have been triggered by late reports from China, Italy, France, Germany, and South Korea indicating higher rates of mortality, as high as 89%, in males compared to females [17]. Infection rates, however, have not widely differed between the sexes [17]. It has been hypothesized that the differences in mortality rate may be due to higher rates of cigarette smoking, alcohol consumption, and the number of pre-existing comorbidities among men compared to women [18]. Lastly, workers or students in the health care sector reported a higher likelihood of acquiring the infection, as well as a higher likelihood of vaccinating, as one might expect. No major differences were noted amongst those with and without underlying illnesses.

### Limitations

One major limitation of this study is its sampling technique. Convenience sampling, typically utilized in cross-sectional studies, is a type of nonprobability sampling that allows for data collection from a group of people who are easy to contact or reach [19]. This may have introduced a sampling bias in our subject cohort. For instance, female and younger subjects are more likely to be represented in social media compared to their male and older counterparts [11], as seen in our study population. Another major limitation is the susceptibility of the study to a nonresponse bias. Our sample also had an unusually high prevalence (22%) of subjects either working or studying in the health care sector. This may be explained by the fact that the survey link was posted on platforms managed by physicians. Such platforms would typically attract users from the same profession. Additionally, the majority of respondents resided in a confined geographical area located within the Arabian Gulf Peninsula. Thus, the data may not be applicable to subjects residing elsewhere. We plan to address such limitations with future studies targeting a wider and more diverse sample of the population.

### Conclusions

Our results are indicative of a well-informed cohort, implementing appropriate protective measures, with the majority reporting social media as their main source of COVID-19 information. This demonstrates that social media is an impactful way of collecting data and delivering information, even though there may be inherent limitations to this study design. We plan to use this platform again to determine if such changes are maintained with the continuation of the pandemic.

## Appendix

Multimedia Appendix 1 Survey questions.

## Abbreviations

CDC: Centers of Disease Control and Prevention
RNA: ribonucleic acid
WHO: World Health Organization
