# Supplementary material for: Perceptions, Knowledge, and Behaviors Related to COVID-19 Among Social Media Users: Cross-Sectional Study
Source: J Med Internet Res. 2020 Sep 8;22(9):e19913. doi: 10.2196/19913 (PMC7481018; doi:10.2196/19913)
Supplement: Multimedia Appendix 1 [file jmir_v22i9e19913_app1.pdf]

COVID-19 Survey

RCSI

استطلاع خاص عن الآراء و الممارسات المتعلقة بفيروس "الكورونا" المستجد

أنت مدعو للمشاركة معنا في استطلاع دراسة مقرر إجرائها من قبل باحثين بالكلية الملكية للجراحين في أيرلندا - البحرين.
الهدف من هذا الاستطلاع هو فهم الآراء و الممارسات الحالية المتعلقة بفيروس "الكورونا" المستجد.

من خلال مشاركتك في الاجابة على هذا الاستبيان، ستساعدنا في فهم الآراء و الممارسات المتعلقة بفيروس الكورونا المستجد بشكل أفضل.

ان اكمال الاستبيان يستغرق 5 دقائق من وقتك. و لا يتعين عليك المشاركة في هذه الاستبيان إذا لم ترغب في ذلك. ولأن السرية والخصوصية هي أولويتنا؛ فان جميع المعلومات التي تدلي بها سيتم الحفاظ عليها بطريقة آمنة. كما أنه اذا كان عمرك 18 عامًا فما فوق، فأنت مؤهل للمشاركة. و اذا احسست بقلق سببه الاستبيان فيمكنك التواصل معنا على covidstudybh@rcsi-mub.com و سنقوم بتوفير مصادر تثقيفية لمساعدتك.

قيامك بالإجابة على أسئلة هذه الدراسة يعتبر موافقة منك على المشاركة في هذه الدراسة البحثية .

Assessment of Public’s Attitudes and Behaviors Towards the Novel Coronavirus COVID-19

You are being asked to participate in a survey conducted by researchers at the Royal College of Surgeons in Ireland – Medical University of Bahrain. This survey is a part of a study done to help us understand what people are thinking about the coronavirus pandemic, and what actions they are taking.

If you wish to take part, this survey will take approximately 5 minutes. The survey is entirely voluntary and completely anonymous – we will not contact you individually after the survey and your answers are confidential. All information will be protected and no one outside this study will have access to your information. There is no cost for participating in this research study, and there is no payment to you, but we are grateful for your help. This study is only for individuals aged 18 years and above.

If you feel distressed or uncomfortable because of the survey please let us know at covidstudybh@rcsi-mub.com and we will provide you with support links. This email will also be provided to you at the end of survey.

By completing the survey, you consent to participate in the study, and you confirm that you are 18 years of age or above.

1- من أين تستقي معلوماتك عن فيروس الكورونا المستجد؟ (اختر كل ما ينطبق)

1. Which of the following sources have you used to learn about the new coronavirus? (Select all that applies)

1.*

Social Media / وسائل التواصل الاجتماعي
Local Newspaper / الجرائد المحلية
TV / التلفاز
Radio / الاذاعة
My Doctor / طبيبي
Close Family / الأقرباء
Coworkers, Classmates / زملاء العمل أو الدراسة
Work / الجهة المسؤولة في العمل
World Health Organization / منظمة الصحة العالمية
Others: / أخرى:

2- أي من هذه الممارسات قمت بها بازدياد في الأسبوع الماضي مقارنة بمرحلة ما قبل فيروس الكورونا ؟ (اختر كل ما ينطبق)

2. Which of the following have you done in the past seven days in addition to what you normally do ? (Select all that applies)

2.*

Washed / Sanitized hands / غسل و تعقيم اليدين
Worked or studied at home / العمل أو الدراسة من المنزل
Visited a doctor / زيارة طبيب
Worn a face mask / لبس الكمام
Stockpiled Food / Water / تخزين المؤونات الغذائية أو الماء
Avoided contact high-risk people / تجنب مخالطة المصابين أو المعرضين للاصابة
Avoided public places / crowds / تجنب الأماكن العامة
Canceled / Postponed social activities / تجنب أو الغاء تجمعات العائلة أو الأصدقاء
Avoided eating at restaurants / تجنب تناول الأكل من المطاعم
Avoided Chinese restaurants / تجنب تناول الأكل من المطاعم الصينية تحديدا
Canceled / Postponed Pleasure Travel / الغاء أو تأجيل سفر لغرض السياحة
Canceled / Postponed Work travel / الغاء أو تأجيل سفر لغرض متعلق بالعمل
Canceled / Postponed Work/School Activities / الغاء أو تأجيل أنشطة العمل أو أنشطة الدراسة
Cancelled a doctor appointment / الغاء موعد طبيب
I have not changed my behaviour / لم أغير شيء

3- "أثرت أزمة الكورونا الحالية سلبياً على حالتي المادية." ما مدى اتفاقك مع هذه العبارة؟

3. "The current coronavirus pandemic has negatively affected my personal finances". Do you agree or disagree with the last statement?

3.*

Agree / أتفق
Disagree / لا أتفق
I do not know / لا أعرف

4- تصور أن اليوم تم توفير لقاح آمن و فعال ضد فيروس الكورونا، ما هي نسبة تقبلك لأخذ اللقاح؟

4. Suppose that a safe and effective coronavirus vaccine was available today. How likely are you to get yourself vaccinated?

4.*

Very Likely / متقبل جداً
Somewhat Likely / متقبل لحد ما
Neutral / محايد
Somewhat Unlikely / غير متقبل لحد ما
Very Unlikely / غير متقبل أبدا

5- هل تم تشخيصك بفيروس الكورونا المستجد؟

5. Have you been diagnosed with the new coronavirus COVID-19?

5.*

Yes / نعم
No / لا

6- اذا كانت اجابتك "لا"، ماهي باعتقادك احتمالية اصابتك بفيروس الكورونا في الثلاثة شهور القادمة؟

6. If you answered “No” to the above question, what do you think is the likelihood you will get the new coronavirus infection in the next 3 months?

6.

Very Likely / احتمالية عالية
Somewhat Likely / احتمالية متوسطة
Somewhat Unlikely / احتمالية قليلة
Very Unlikely / احتمالية غير واردة أبدا
I do not know / لا أعرف

7- ما هي باعتقادك أهم أعراض الاصابة بفيروس الكورونا؟ (اختر كل ما ينطبق)

7. Which of the following are the main symptoms people infected with the coronavirus (COVID-19) experience (Select all that applies)?

7.*

Fever or chills / ارتفاع حرارة الجسم
Runny or stuffy nose / الزكام
Skin rash / طفح جلدي
Cough / السعال
Sore throat / احتقان بالبلعوم
Chest congestion / احتقان بالصدر
Muscle or body aches / الام بالعضلات أو الجسم
Headaches / صداع
Fatigue or tiredness / تعب و ارهاق
Shortness of breath / ضيق التنفس
Abdominal discomfort / الام بالبطن
I don't know / لا أعرف

8- هل تعاني من أي من المشاكل الصحية التالية؟ (اختر كل ما ينطبق)

8. Do you have any of the following medical problems? (Select all that applies)

8.*

High Blood Pressure / ارتفاع ضغط الدم
Diabetes / السكري
Heart Disease / أمراض القلب
Lung Disease / أمراض رئوية
Cancer / أمراض السرطان
Others: / أخرى
I have no medical problems / ليس لدي أي مشاكل صحية

9- العمر:

9. How old are you?

9.*

18-24
25-34
35-44

45-54
55-64
65 and above / 65 و فوق

10- الجنس:

10. What is your gender?
10.*
Female / أنثى
Male / ذكر

11- أعلى مستوى تعليمي:

11. What is your highest educational level?
11.*
Primary School / ابتدائي
Intermediate School / اعدادي
High School / ثانوي
College / Higher Education Degree / شهادة جامعية

12- هل تعمل أو تدرس حاليا بالقطاع الصحي؟

12. Do you currently work or study in the healthcare sector?
12.*
Yes / نعم
No / لا

13- بأي دولة تقيم حاليا؟

13. Which country do you currently reside in?
13.*
-Select-

شكرا لك على مساهمتك في هذا الاستبيان. بالنسبة لأية أسئلة أو استفسارات تهمك بخصوص هذه الدراسة، فيرجى التواصل على covidstudybh@rcsi-mub.com

Thank you for participating, if you have any questions about the study, please email covidstudybh@rcsi-mub.com
